# Effects of ferulic acid on growth performance and intestinal oxidation indexes of Jilin white geese under lipopolysaccharide-induced oxidative stress

**DOI:** 10.1371/journal.pone.0291955

**Published:** 2023-10-12

**Authors:** Yingkun Liu, Tao Zhang, Fangyuan Jia, Haojia Li, Meng Sun, Zengyu Fu, Haizhu Zhou, Wei Guo, Yunhang Gao

**Affiliations:** 1 Jilin Agricultural University, Changchun, Jilin, China; 2 Jilin Provincial Science and Technology Innovation Platform Management Center, Changchun, Jilin, China; 3 Jilin Animal Husbandry Station, Changchun, Jilin, China; Benha University, EGYPT

## Abstract

In geese breeding, due to the frequent influence of drugs and environmental and other factors, geese are extremely prone to oxidative stress, which adversely affects growth and development, geese meat quality, down production, and severely affects the development of the geese industry. Ferulic acid from plant extracts can be used as a feed additive, which is safe and non-toxic, and it can exert certain therapeutic effects on oxidative stress in geese. This experiment investigated the effect of ferulic acid on the growth performance, organs indices, and intestinal oxidative indices of Jilin white geese under lipopolysaccharide-induced oxidative stress. Geese were randomly divided into six groups: C (blank control), L (lipopolysaccharide control), F1 (60 mg/kg ferulic acid), F2 (120 mg/kg ferulic acid), F3 (180 mg/kg ferulic acid), and F4 (240 mg/kg ferulic acid). Groups L and F1–F4 were injected intraperitoneally with 0.5 mg/kg lipopolysaccharide and group C with an equivalent volume of normal saline on days 14,17 and 20, and 10 animals from each group were randomly selected for slaughter on day 21. The results showed that: 1) On day 14, the final body weight and average daily feed intake were significantly higher in group F3 than in group L, and on day 21, the final body weight was significantly higher in group F3 than in group L. 2) The thymus index was significantly higher in group F4 than in group L. 4) In the duodenum, MDA activity was reduced in group C compared with that in group L. 5) In the jejunum and ileum, MDA was significantly lower in group F3 than in group L. These results show that the addition of 180 mg/kg of ferulic acid to the diet can promote the growth of geese and alleviate the damage caused by oxidative stress in all intestinal segments.

## Introduction

Ferulic acid (FA) is a phenolic substance and an important active ingredient that is common in various plants. It occurs at high concentrations in food ingredients such as coffee, grain hulls, vanilla beans, wheat bran, and rice bran [1,2]. The FA molecule is a 4-hydroxy-3-methoxy cinnamic acid (C_10_ H_10_ O_4_), and FA exhibits various biological activities and physiological functions and has low toxicity [3,4]. Importantly, FA has been shown to exhibit antioxidant and anti-inflammatory properties and is an effective modulator of multidrug resistance in cancer [5]. FA can prevent acute liver injury due to sepsis by attenuating the inflammatory response, and it improves corticosterone-induced liver damage [6]. Moreover, FA also improves corticosterone-induced depressive behaviour and oxidative stress in mice [7], and enhances the antibacterial activity of quinolone antibiotics [8]. Geese is a nutritious and healthy food resource. the short reproductive periods, low hatchability, and high embryo mortality of geese [9]. it is reported that the content of protein and trace elements in the meat of goose is higher than in other poultry products [10]. So, geese breeding industry has broad prospects. Intestine plays a key supporting role in the growth of animals. and early growth and development of the gastrointestinal tract are critical to optimizing the growth of poultry [11]. In the early stages of goose growth and development, a large amount of nutritional support is required. After the intestinal structure and function are damaged, it will affect the individual’s early nutritional absorption, causing irreversible damage to the body’s growth and development in the early stages[12].

Lipopolysaccharide (LPS) is a pathogenic compound that occurs in the outer membrane of the cell wall of all gram-negative bacteria, and it can elicit multiple signaling events in the cell [13]. LPS can act as a strong inflammatory mediator and is widely used in animal studies to simulate bacterial infection [14]. Peng et al.[15] studied inflammation in bovine endometrial epithelial cells caused by LPS and found that FA exerted an anti-inflammatory effect by inhibiting the release of cytokines. Chen et al [16]. reported that 30 mg/kg FA increased the antioxidant capacity of the liver and repaired liver damage, reducing hepatocyte death due to LPS in blunt-nosed sea bream. Further, FA plays a positive role in reducing renal injury in HFD/STZ-induced DN mice by enhancing autophagy and inhibiting inflammation [17].

However, to date, the effects of FA treatment in geese after triggering oxidative stress have not been studied, nor has the oxidative stress damage encountered during the development of the geese industry been addressed, which would lead to mortality in geese, and could adversely affect the development of the geese industry. In the present study, FA was added to the diets of Jilin white geese at different concentrations, and LPS was injected intraperitoneally at 14 and 21 days of age to investigate the effects of FA on the growth performance and intestinal antioxidant capacity of LPS-stressed Jilin white geese. Our findings show that FA can protect animals under oxidative stress conditions and provide a scientific basis for the implementation of FA as an antioxidant agent.

## Materials and methods

### Experimental design

This study was carried out in strict accordance with the recommendations of the Guide to Nursing and Use of Experimental Animals of Jilin Agricultural University. The study was verbal approval by the Experimental Ethics Committee of Jilin Agricultural University. All operations were performed under pentobarbital sodium anesthesia, and every effort was made to reduce pain.120 male Jilin white geese of similar weight at 7 d of age were used. There was a pre-feeding period of 7 d, and the trial period was 21 d. After the pre-feeding period, the 120 Jilin white geese were randomly divided into six groups with five replicates (four birds in each group). Groups F1 (60 mg/kg feed FA), F2 (120 mg/kg feed FA), F3 (180 mg/kg feed FA), F4 (240 mg/kg feed FA), and L were given intraperitoneal injections of LPS (500 μg/kg BW) on days 14, 17, and 20. The doses and routes of LPS administration referred to the previous studies [18,19]. Group C was given intraperitoneal injections of normal saline (0.5 mg/kg BW). The test was carried out by establishing an oxidative stress model. Table 1 shows the composition of rations. During the test period, all geese had access to feed and water ad libitum throughout the trial. Water was provided in a half-open plastic cylindrical water tank, and the feed was provided in feeders on one side of each pen. The geese were reared indoors conditions (temperature: 26.0°C ± 3.0°C; relative humidity (RH): 60.5 ± 5.0%; lighting period: 16 h; space allocation: 0.49 m2/gander), and the feed intake and body weight were recorded daily. On day 21, 10 animals in each group were randomly selected for slaughter, and tissue samples of the heart, liver, spleen, kidney, bursa of fabricius, and thymus organs, duodenum, jejunum, and ileum were collected.

**Table 1:** Basic feed composition and nutrient level (air dry basis) %.

Ingredient (%)		Nutritional level	
Corn	53.30	Metabolic energy (MJ/kg)	10.96
Soybean meal	22.00	Crude protein (%)	14.56
Corn protein flour	3.00	Crude fiber (%)	4.02
Corn stalks	8.00	Neutral detergent fiber (%)	20.18
Cottonseed oil	4.00	Acid detergent fiber (%)	12.16
fish meal	5.50	Methionine (%)	0.47
Premix^1)^	1.00		
Limestone	1.70		
CaHPO_4_	1.00		
NaCl	0.50		
Total	100.00		

1) The premix provided the following per kg of diets: VA 2500 IU, VD_3_ 1000 IU, VE 3100 mg, VK_3_ 200 mg, VB_1_ 100 mg, VB_2_ 1 200 mg, VB_6_ 200 mg, VB_12_ 2 mg, Nicotinic acid 600 mg. Pantothenic acid 1 700 mg, Folic acid 200 mg, Biotin 20 mg, Fe (as ferrous sulfate) 6 000 mg, Cu (as copper sulfate) 300 mg, Mn (as manganese sulfate) 15 000 mg. Zn (as zinc sulfate) 8 500 mg, I (as potassium iodide) 10 mg, Se (as sodium selenite) 30 mg.

### Test materials

#### Test animals and reagents

The test animals were purchased from Jilin Yuhong Ecological Agriculture Technology Co. We also used *E*. *coli* lipopolysaccharide (Sigma Chemical Co., St. Louis, MO, USA), FA (97%) (Shanghai Maclean Biochemical Technology Co., Ltd), and Malondialdehyde assay (MDA)kit, Total Antioxidant Dismutase Assay (SOD) Kit, Glutathione peroxidase assay (GSH-PX) kit, Hydrogen peroxidase assay (CAT) kit, Total Antioxidant Capacity Assay(T-AOC) Kit (Nanjing Jiancheng Bioengineering Institute, Nanjing, China).

### Sample collection and indicator determination

Growth performance. The initial weight, day 14 weight, and final weight of the geese were recorded. The average daily gain (ADG), average daily feed intake (ADFI), and feed conversion ratio (F/G) in the stages of days 1–14, 15–21, and 1–21 in the trial were calculated.

#### Visceral index

All surgeries are performed under pentobarbital sodium anesthesia, and every effort was made to reduce pain. Slaughter was carried out on day 21 of the trial. The organs of the geese (heart, liver, spleen, bursa, and thymus) were removed after slaughter and were weighed after removing excess fat.

Measurement of intestinal oxidative stress indicators. Determination of MDA, SOD, GSH-PX, CAT and T-AOC in the intestinal tract of geese.

### Data analysis

The raw data were processed using Microsoft Excel. A one-way ANOVA and Duncan’s multiple comparison test were performed using SPSS 26.0 software. Results are presented as mean ± standard error (SE), with *P <* 0.05 indicating a significant difference.

## Results

### Growth performance

The LPS damage model was developed to observe the change in growth performance of each group before and after oxidative stress (Table 2). Between days 1 and 14, final body weight (FB) and ADG were significantly higher in group F3 than in group L (*P <* 0.05), and ADFI was significantly higher in groups F2 and F3 than in groups C and L (*P* < 0.05). The FCR was significantly lower in group F3 than in group L (*P* < 0.05). On days 15–21, FB was significantly higher in the groups with FA added than in group L (*P* < 0.05). ADG was significantly higher in the groups with FA added than in group L and significantly lower than in group C (*P* < 0.05). ADFI was significantly higher in the groups with F1 and F4 than in group L and significantly lower in the groups with FA added and L than in group C (*P* < 0.05). FCR was significantly lower in the groups with FA added than in group L and significantly higher in groups F1, F2, F3, and L than in group C (*P <* 0.05). From days 1 to 21, ADG was significantly higher in the groups with FA added than in group L and significantly lower in groups F1 and L than in group C (*P <* 0.05). ADFI was significantly higher in groups C, F2, and F3 than in group L (*P <* 0.05). FCR was significantly higher in group L than in the other groups (*P <* 0.05).

**Table 2:** Effect of FA on the growth performance of Jilin white geese.

Time	Items	Groups					
		L	C	F1	F2	F3	F4
Days 1–14	IB/g	1212.00±78.80	1221.80±80.40	1245.20±8.52	1230.40±89.35	1219.60±31.62	1257.20±64.33
	FB/g	1697.00±46.33^b^	1727.40±44.73^ab^	1762.40±15.57^ab^	1760.40±59.40^ab^	1837.20±21.62^a^	1744.00±32.81^ab^
	ADG/g	34.64±4.94^b^	36.11±2.94^ab^	36.64±1.30^ab^	37.85±2.63^ab^	44.11±1.30^a^	34.77±2.46^b^
	ADFI/g	194.10±1.07^b^	193.47±1.68^b^	189.96±1.89^b^	199.07±0.40^a^	198.59±0.86^a^	192.33±2.28^b^
	FCR	6.17±0.93^a^	5.50±0.41^ab^	5.15±0.17^ab^	5.24±0.30^ab^	4.52±0.13^b^	5.67±0.38^ab^
Days 15–21	FB/g	1866.20±49.19^c^	2267.80±26.62^a^	2094.40±15.34^b^	2144.40±47.38^ab^	2209.80±58.23^ab^	2152.80±43.07^ab^
	ADG/g	24.17±1.50^c^	77.20±3.34^a^	47.43±2.21^b^	54.86±4.30^b^	53.23±6.06^b^	58.40±3.89^b^
	ADFI/g	196.70±5.20^a^	239.29±0.77^c^	220.49±4.38^b^	226.04±4.29^ab^	229.29±4.87^ab^	221.89±7.97^b^
	FCR	8.30±0.52^a^	3.12±0.13^c^	4.67±0.19^b^	4.21±0.36^b^	4.44±0.41^b^	3.76±0.30^bc^
Days 1–21	ADG/g	31.15±3.45^c^	49.81±2.59^a^	40.44±1.09^b^	43.52±3.13^ab^	47.15±1.59^ab^	42.65±1.57^ab^
	ADFI/g	194.97±1.81^b^	208.74±4.96^a^	200.14±3.72^ab^	208.06±3.16^a^	208.82±3.63^a^	202.18±4.28^ab^
	FCR	6.58±0.76^a^	4.21±0.32^b^	5.08±0.28^b^	4.80±0.34^b^	4.52±0.33^b^	4.82±0.41^b^

Results are presented as means ± SE (n = 10); values in the same row without letters or with the same letter superscript indicate no significant difference, while different small letter superscripts indicate a significant difference (at *P* < 0.05); Group L = 0 mg/kg FA+0.5 mg/kg LPS; Group C = 0 mg/kg FA+0.5 mg/kg NS; Group F1 = 60 mg/kg FA + 0.5 mg/kg LPS; Group F2 = 120 mg/kg FA + 0.5 mg/kg LPS; Group F3 = 180 mg/kg FA + 0.5 mg/kg LPS; Group F4 = 240 mg/kg FA + 0.5 mg/kg LPS; IB = initial body weight; FB = final body weight; ADG = daily weight gain; ADFI = average daily feed intake; FCR = feed to meat ratio.

### Organs indices

To reflect the changes in organ weight in geese after oxidative stress, samples were taken at 21 days and the organs indices was measured (Table 3). The thymic indices was significantly higher in group F4 than in groups C (*P <* 0.05).

**Table 3:** Effect of FA on organs indices of Jilin White Geese.

Items	Groups					
	L	C	F1	F2	F3	F4
Heart indices (%)	0.79±0.07	0.73±0.03	0.68±0.07	0.88±0.17	0.83±0.12	0.76±0.06
Liver indices (%)	2.92±0.05	3.05±0.14	2.61±0.11	2.98±0.26	3.00±0.16	3.19±0.49
Spleen indices (%)	0.11±0.01	0.13±0.02	0.12±0.01	0.10±0.02	0.12±0.02	0.15±0.02
Bursa indices (%)	2.62±0.10	2.45±0.17	2.48±0.24	2.91±0.06	2.55±0.25	2.87±0.30
Thymus indices (%)	0.12±0.02^bc^	0.10±0.01^c^	0.21±0.05^ab^	0.13±0.03^abc^	0.15±0.03^abc^	0.18±0.02^ab^

Results are expressed as means ± SE (n = 10); values in the same row without letters or with the same letter superscript indicate no significant difference, while different small letter superscripts indicate a significant difference (at *P* < 0.05); L = LPS Jilin white geese oxidative stress group; C = Blank control group without any addition; Group L = 0 mg/kg FA+0.5 mg/kg NS; group C = 0 mg/kg FA + 0.5 mg/kg LPS; Group F1 = 60 mg/kg FA + 0.5 mg/kg LPS; Group F2 = 120 mg/kg FA + 0.5 mg/kg LPS; group F3 = 180 mg/kg FA + 0.5 mg/kg LPS; group F4 = 240 mg/kg FA + 0.5 mg/kg LPS.

### Antioxidant activity in the duodenum

We measured the changes in antioxidant activity in the duodenum of geese after oxidative stress to reflect the antioxidant activity of the duodenum (Table 4). Groups F1, F3, F4, and C showed a significant decrease in MDA compared to group L (*P <* 0.05). Group F1 showed a significant decrease in SOD compared to group C and Group F4 showed a significant reduce in GSH-Px compared to group C (*P <* 0.05). Group C showed a significant increase in SOD and GSH-Px compared to group L (*P <* 0.05). Groups L, F1, F2, and F4 showed a significant decrease in CAT compared to group C (*P <* 0.05).

**Table 4:** Effect of ferulic acid on duodenal intestinal antioxidant capacity of Jilin White Geese.

Items	Groups					
	L	C	F1	F2	F3	F4
MDA/(nmol/mg)	34.37±6.45^b^	17.84±2.10^a^	17.95±2.33^a^	25.60±4.53^ab^	19.22±0.93^a^	19.44±2.59^a^
SOD/(U/mg)	0.33±0.03^b^	0.45±0.02^a^	0.31±0.01^b^	0.37±0.03^ab^	0.35±0.04^ab^	0.41±0.05^ab^
GSH-Px/(U/mg)	32.88±2.09^b^	51.38±2.04^a^	42.62±7.77^ab^	41.87±4.59^ab^	46.13±3.76^ab^	34.00±1.96^b^
CAT/(U/g)	4.16±0.24^b^	6.13±0.58^a^	4.23±0.66^b^	4.44±0.11^b^	5.14±0.72^ab^	4.91±0.17^b^
T-AOC/(mmol/g)	0.39±0.27	0.61±0.16	0.23±0.01	0.28±0.01	0.49±0.20	0.60±0.17

Results are expressed as means ± SE (n = 10); values in the same row without letters or with the same letter superscript indicate no significant difference, while different small letter superscripts indicate a significant difference (at *P* < 0.05); L = LPS Jilin white geese oxidative stress group; K = Blank control group without any addition; Group L = 0 mg/kg FA+0.5 mg/kg NS; group C = 0 mg/kg FA + 0.5 mg/kg LPS; Group F1 = 60 mg/kg FA + 0.5 mg/kg LPS; Group F2 = 120 mg/kg FA + 0.5 mg/kg LPS; group F3 = 180 mg/kg FA + 0.5 mg/kg LPS; group F4 = 240 mg/kg FA + 0.5 mg/kg LPS; MDA = malondialdehyde; SOD = superoxide dismutase; GSH-Px = glutathione peroxidase; CAT = catalase; T-AOC = total antioxidant capacity.

### Antioxidant activity in the jejunum

We measured the changes in antioxidant activity in the jejunum of geese after oxidative stress to reflect the antioxidant activity in the duodenum (Table 5). MDA was significantly lower in groups F3 and F4 than in group L and in group F4 than in group F1 (*P <* 0.05). SOD was significantly higher in group C than in groups L and F1 (*P <* 0.05). GSH-Px was significantly higher in group F3 than in groups L, C, and F1 (*P <* 0.05). The CAT content of group L was significantly lower than that of all other groups (*P <* 0.05).

**Table 5:** Effect of ferulic acid on jejunal antioxidant capacity of Jilin White Geese.

Items	Groups					
	L	C	F1	F2	F3	F4
MDA/(nmol/mg)	45.47±5.77^a^	19.73±2.66^c^	38.33±8.27^ab^	30.47±1.79^abc^	27.67±7.27^bc^	20.20±2.27^c^
SOD/(U/mg)	70.61±3.62^b^	95.49±9.24^a^	74.49±6.97^b^	76.64±7.62^ab^	84.29±1.99^ab^	88.31±3.69^ab^
GSH-Px/(U/mg)	0.50±0.03^b^	0.60±0.03^b^	0.58±0.24^b^	0.73±0.07^ab^	1.07±0.19^a^	0.69±0.02^ab^
CAT/(U/g)	0.53±0.14^b^	2.39±0.16^a^	2.58±0.70^a^	3.03±0.68^a^	3.33±0.43^a^	2.53±0.28^a^
T-AOC/(mmol/g)	0.17±0.04	0.31±0.07	0.26±0.05	0.30±0.06	0.32±0.02	0.27±0.06

Results are expressed as means ± SE (n = 10), where values in the same row without letters or with the same letter superscript indicate no significant difference (*P*>0.05), while different small letter superscripts indicate a significant difference (*P*<0.05); L = LPS Jilin white geese oxidative stress group; K = Blank control group without any addition; Group L = 0 mg/kg FA+0.5 mg/kg NS; group C = 0 mg/kg FA + 0.5 mg/kg LPS; Group F1 = 60 mg/kg FA + 0.5 mg/kg LPS; Group F2 = 120 mg/kg FA + 0.5 mg/kg LPS; group F3 = 180 mg/kg FA + 0.5 mg/kg LPS; group F4 = 240 mg/kg FA + 0.5 mg/kg LPS; MDA = malondialdehyde; SOD = superoxide dismutase; GSH-Px = glutathione peroxidase; CAT = catalase; T-AOC = total antioxidant capacity.

### Antioxidant activity in the ileum

We measured the changes in antioxidant activity in the jejunum of geese after oxidative stress to reflect the antioxidant activity in the duodenum (Table 6). MDA was significantly lower in groups F2, and C than in group L, (*P <* 0.05). GSH-Px was significantly higher in group C than in groups L and F1, F2, F4 and significantly higher in group F2, F3, F4 than in group L and significantly higher in group F3 than in group F1 (*P* < 0.05).

**Table 6:** Effect of ferulic acid on ileum antioxidant capacity of Jilin White Geese.

Items	Groups					
	L	C	F1	F2	F3	F4
MDA/(nmol/mg)	22.46±0.91^a^	14.36±1.08^b^	16.62±2.28^ab^	15.55±0.78^b^	16.79±1.57^ab^	18.69±3.43^ab^
SOD/(U/mg)	91.64±11.40	120.28±10.25	101.10±7.03	103.79±8.76	108.95±2.34	101.64±9.67
GSH-Px/(U/mg)	38.79±4.75^d^	59.10±1.47^a^	44.97±2.52^cd^	48.24±1.64^bc^	55.04±1.50^ab^	48.93±0.91^bc^
CAT/(U/g)	6.39±0.14	7.61±0.09	6.68±0.12	7.19±1.49	7.61±0.30	7.37±0.25
T-AOC/(mmol/g)	0.07±0.02	0.20±0.04	0.15±0.01	0.23±0.04	0.25±0.06	0.18±0.06

Results are expressed as Mean±SE (n = 10), where values in the same row without letters or with the same letter superscript indicate no significant difference (*P*>0.05), while different small letter superscripts indicate a significant difference (*P*<0.05); L = LPS Jilin white geese oxidative stress group; K = Blank control group without any addition; Group L = 0 mg/kg FA+0.5 mg/kg NS; group C = 0 mg/kg FA + 0.5 mg/kg LPS; Group F1 = 60 mg/kg FA + 0.5 mg/kg LPS; Group F2 = 120 mg/kg FA + 0.5 mg/kg LPS; group F3 = 180 mg/kg FA + 0.5 mg/kg LPS; group F4 = 240 mg/kg FA + 0.5 mg/kg LPS; MDA = malondialdehyde; SOD = superoxide dismutase; GSH-Px = glutathione peroxidase; CAT = catalase; T-AOC = total antioxidant capacity.

## Discussion

During the process of goose breeding, oxidative stress can lead to a decrease in gse growth performance and a loss of economic benefits. LPS, a major component of the cell wall of gram-nega-tive bacteria, is a pathogenic compound [20]. In this study, we obtained equivalent experimental results: LPS injection at 0.5 mg/kg body weight significantly reduced both the body weight gain of geese at d 15 to 21 and the feed intake during, and, but increased the FCR of geese on d 15 to 21. These initial results indicated that the oxidative stress model was established successfully and was suitable for subsequent investigation of the effects of FA on geese health. The addition of 6 mg/kg of FA to the feed increased beef the tenderness, juiciness, flavor intensity, and amount of some fatty acids [21]. Supplementation with 180 mg/kg of FA in the present study enhanced growth performance and reversed the decline in growth performance brought about by oxidative stress, indicating that ferulic acid can alleviate the damage caused by oxidative stress.

The liver, as the main metabolic and detoxification organ in the body, is closely related to various physiological functions and circulation, and the effects of immune organs such as the liver on oxidative stress occur through the recruitment of immune cells [22]. In the present study, the addition of 240 mg/kg FA increased the thymic index, probably because FA mitigates the effects of oxidative stress from LPS by affecting the weight of the thymus and increasing body immunity.

Reported that FA has cytoprotective capacity and may promote gastrointestinal health and microbial protein synthesis [23]. In mice with high oxidative stress levels, insulin synthesis was improved in pancreatic β-cells [24]. In the present study, the addition of 180 mg/kg of FA significantly alleviated the intestinal damage caused by oxidative stress, may be related to the enhancement of intestinal epithelial cell activity by ferulic acid, enhances cell activity to resist LPS induced damage.

FA also inhibited the activation of the ROCK/NF-κB signaling pathway, thereby improving the dysregulation of oxidative stress and inflammation and exerting an effective hepatoprotective effect [25]. The above growth performance and intestinal oxidative indexes were due to excessive reactive oxygen species production from LPS-induced oxidative stress, which indirectly affected the changes in physiological indexes and enzyme activities. This may be related to antioxidant signaling pathways, such as Nrf2/HO-1 and ROCK/NF-κB. Administering FA after LPS-induced oxidative stress can alleviate the effects of oxidative stress on growth performance and reduce intestinal antioxidant activity.

## Conclusion

1. Adding 180 mg/kg of FA can increase the body weight of geese and promote their growth. Adding 60 mg/kg FA can improve the thymus index, alleviate the damage to immune function caused by stress, and reduce the negative effects of stress.

2. Adding 180 mg/kg FA can alleviate oxidative stress damage in the duodenum, ileum, and jejunum, reduce cell membrane damage, maintain the homeostasis of the membrane lipid bilayer, and protect cells from oxygen ions.

In conclusion, adding 180 mg/kg of FA promoted the growth of geese and alleviated the effects of oxidative stress and the damage caused by oxidative stress in the duodenum, jejunum, and ileum.
